# A Simple Hemorrhagic Hepatic Cyst: Warfarin Mediated

**DOI:** 10.14309/crj.0000000000000567

**Published:** 2021-04-26

**Authors:** Piruthiviraj Natarajan, Sudhagar Thangarasu, Vamsi Krishna Kunam, Priyadarshini Loganathan, Swaytha Ramaraj Ganesh, Mahesh Gajendran

**Affiliations:** 1 Department of Internal Medicine-Transmountain, Texas Tech University Health Sciences Center El Paso, El Paso, TX; 2 Department of Radiology, Texas Tech University Health Sciences Center El Paso, El Paso, TX; 3 Department of Gastroenterology and Hepatology, University of Pittsburgh Medical Center, Pittsburgh, PA

## CASE REPORT

**Figure 1.**
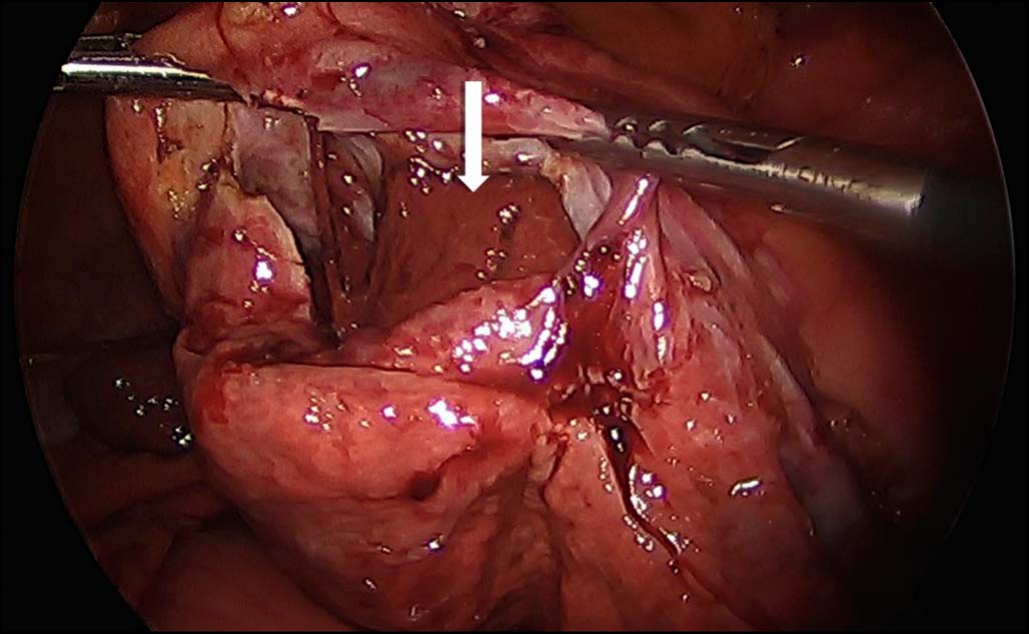
Axial contrast-enhanced computed tomography image showed large, expansile cystic lesion (arrows) replacing the left lobe of the liver with mild heterogeneous attenuation and thin internal septation (arrow heads).

**Figure 2.**
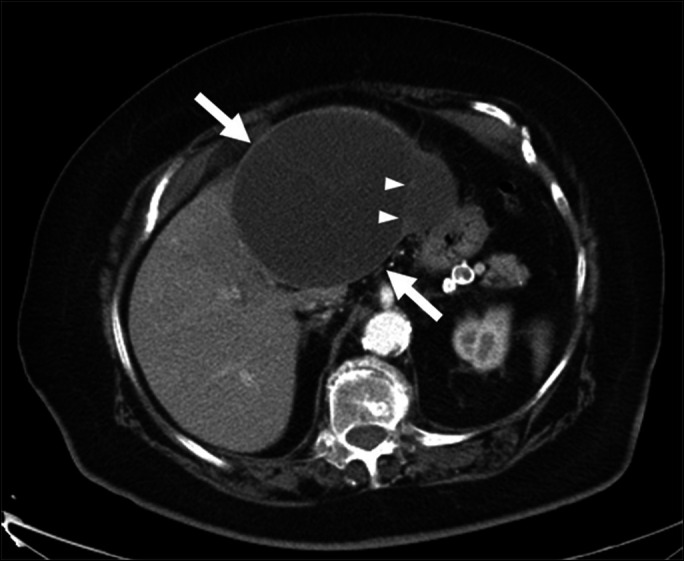
Intraoperative image—hepatic cyst filled with bile and blood.

A 66-year-old woman with a medical history of hypertension, hyperlipidemia, and pulmonary embolism on warfarin therapy presented with acute right upper quadrant abdominal pain ongoing for 4 weeks. On physical examination, tenderness was elicited in the right upper quadrant without rebound or guarding. Laboratory testing revealed an international normalized ratio of 3.2 and normal liver chemistries. An abdominal computed tomography (CT) showed a 15-cm expansile cystic lesion replacing the left lobe of the liver with mild heterogeneous attenuation and thin internal septations (Figure 1). Because of ongoing concerns for malignant transformation, a laparoscopic left hepatic segmentectomy with cyst resection was performed. The cyst contained about 1.5 L of bile and blood (Figure 2). Histopathologic evaluation revealed a benign hepatic cyst with chronic inflammation, organized hemorrhage, hemosiderin encrustation, and calcification with surrounding normal liver parenchyma.

Simple hepatic cysts are believed to arise from congenital bile duct malformations that fail to communicate with the biliary tree.^1^ The prevalence of simple hepatic cysts ranges between 5% and 15% depending on the imaging modality and increases with age. Hemorrhagic hepatic cyst (HHC) is a rare complication of simple cysts, with less than 40 cases reported thus far.^2^ The clinical presentation is not a reliable indicator of active bleeding in simple hepatic cyst. However, it is essential to consider hemorrhagic rupture when patients present with severe abdominal pain. Abdominal ultrasound findings such as hyperechogenic septa, a thick wall, and mural nodules suggest hemorrhage.^3^ Accurate diagnosis of the primary etiology of the HHC by imaging studies is challenging because cystic neoplasms such as biliary cystadenomas or cystadenocarcinomas share the same findings as HHC.^4^ Magnetic resonance imaging shows hemorrhage as high-intensity areas on T1- and T2-weighted sequences without enhancement of mural nodules after contrast.^5^ CT scan showed heterogeneous attenuation raising concerns for malignancy; hence, the patient was offered surgical resection. The surgical treatment of choice for HHC is tailored toward patient preference and eligibility. Percutaneous aspiration of the cyst is less invasive but has a higher risk of recurrence and infection. Sclerotherapy is being used more and has gained traction because of reduced recurrence. Cyst resection is preferred over wide unroofing/fenestration for suspected hemorrhagic contents. In conclusion, hemorrhagic features on imaging are considered a “red flag” for malignancy, which can only be definitively ruled out with a surgical biopsy.

## DISCLOSURES

Author contributions: P. Natarajan drafted the manuscript and approved the final manuscript. S. Thangarasu, VK Kunam, P. Loganathan, and SR Ganesh revised the manuscript for intellectual content and approved the final manuscript. M. Gajendran wrote the manuscript, revised the manuscript for intellectual content, and approved the final manuscript. M. Gajendran is the article guarantor.

Acknowledgments: The authors thank P. Estrada, MD, and L. Ruck, MD, for their time and efforts to assist with the revision of the manuscript.

Financial disclosure: None to report.

Informed consent was obtained for this case report.
